# Cerebrospinal Fluid Interleukin-6 in Central Nervous System Inflammatory Diseases

**DOI:** 10.1371/journal.pone.0072399

**Published:** 2013-08-27

**Authors:** Alexandre Wullschleger, Viktoria Kapina, Nicolas Molnarfi, Delphine S. Courvoisier, Jörg D. Seebach, Marie-Laure Santiago-Raber, Denis F. Hochstrasser, Patrice H. Lalive

**Affiliations:** 1 Department of Clinical Neurosciences, Division of Neurology, University Hospital of Geneva, Geneva, Switzerland; 2 Department of Pathology and Immunology, Faculty of Medicine, University of Geneva, Geneva, Switzerland; 3 Division of Clinical Epidemiology, University Hospitals of Geneva, Geneva, Switzerland; 4 School of Public Health, Harvard University, Boston, Massachusetts, United States of America; 5 Department of Internal Medicine, Division of Clinical Immunology and Allergology, University Hospital and Medical Faculty, Geneva, Switzerland; 6 Department of Genetics and Laboratory Medicine, Laboratory Medicine Service, University Hospital of Geneva, Geneva, Switzerland; Klinikum rechts der Isar der Technischen Universitaet Muenchen, Germany

## Abstract

**Background:**

Interleukin (IL)-6 is recognised as an important cytokine involved in inflammatory diseases of the central nervous system (CNS).

**Objective:**

To perform a large retrospective study designed to test cerebrospinal fluid (CSF) IL-6 levels in the context of neurological diseases, and evaluate its usefulness as a biomarker to help discriminate multiple sclerosis (MS) from other inflammatory neurological diseases (OIND).

**Patients and Methods:**

We analyzed 374 CSF samples for IL-6 using a quantitative enzyme-linked immunosorbent assay. Groups tested were composed of demyelinating diseases of the CNS (DD, n = 117), including relapsing-remitting MS (RRMS, n = 65), primary progressive MS (PPMS, n = 11), clinically isolated syndrome (CIS, n = 11), optic neuritis (ON, n = 30); idiopathic transverse myelitis (ITM, n = 10); other inflammatory neurological diseases (OIND, n = 35); and non-inflammatory neurological diseases (NIND, n = 212). Differences between groups were analysed using Kruskal−Wallis test and Mann−Whitney U-test.

**Results:**

CSF IL-6 levels exceeded the positivity cut-off of 10 pg/ml in 18 (51.4%) of the 35 OIND samples, but in only three (3.9%) of the 76 MS samples collected. CSF IL-6 was negative for all NIND samples tested (0/212). IL-6 cut-off of 10 pg/ml offers 96% sensitivity to exclude MS.

**Conclusion:**

CSF IL-6 may help to differentiate MS from its major differential diagnosis group, OIND.

## Introduction

Multiple sclerosis (MS) is an autoimmune disease of multifactorial origin that affects the central nervous system (CNS). [1] Its pathology is characterised by a combination of inflammation, demyelination, and axonal damage. [2] These processes are not uniformly represented across patient populations, but can predominate selectively in individual patients, contributing to the heterogeneity of phenotypic expression of the disease. [3] Due to its complex nature, there is probably no single protein that can serve as a biomarker for MS in a clinically relevant way. However, individuals with MS might be differentiated from those with other inflammatory neurological diseases (OIND), non-inflammatory neurological diseases (NIND) and healthy controls (HC) using a distinct set of individual proteins that are not independently reliable indicators of disease state.

Cerebrospinal fluid (CSF) is a promising body fluid for the search of biomarkers associated with chronic neurological disease. [4], [5] Because the CSF compartment is in close anatomical contact with the brain interstitial fluid, biochemical changes related to MS may be reflected in the CSF. [6], [7] Among mediators recognised to contribute to inflammatory processes in the CNS, accumulating evidence indicates that interleukin-6 (IL-6) is a potential marker of CNS diseases. IL-6 is a pleiotropic glycoprotein cytokine reported to mediate signal transduction between immune cells, [8] and is considered a “double-edged sword” mediator because it has both pro-inflammatory and potentially neuroprotective functions. [9] Elevated IL-6 levels have recently been reported in the CSF of patients with inflammatory CNS diseases, such as idiopathic transverse myelitis (ITM), [10] acute disseminated encephalomyelitis (ADEM), [11] and neuromyelitis optica (NMO), [12] indicating the possible role of IL-6 as a biomarker of inflammatory CNS conditions. The clinical recognition of CSF IL-6 as biomarker of distinct CNS conditions has not yet been clearly demonstrated, partly because of the lack of large cohorts to sufficiently power the studies.

The recently revised 2010 MS criteria have been simplified to allow easier diagnosis of MS without losing sensitivity or specificity. [13] These new criteria also emphasize the importance of excluding other conditions that may mimic MS. As a large number of diseases can mimic brain lesion changes that are seen in MS, it is therefore important to exclude these conditions with appropriate clinical and laboratory investigations before making a definite diagnosis of MS. In this regard, new biomarkers that may help to discriminate between MS and other closely related CNS diseases are mandatory.

In the present study, we investigated the potential of CSF IL-6 as a biomarker of CNS inflammatory diseases by evaluating its levels in a large number of patients with a variety of CNS disorders, primarily to differentiate MS from other inflammatory conditions that may mimic MS.

## Patients and Methods

### Ethics Statement

This study was performed on cerebrospinal fluids (CSF) collected in excess during regular spinal taps that were stored anonymously in a centralized biobank for retrospective research purposes. This research was conducted accordingly to the regulations of the Act on Medical Research Involving Human Subjects of the Geneva University Hospital (HUGO.RE.DG.0010). Neither written nor recorded verbal informed consent was compulsory for this research study involving the retrospective analysis of fluid collection, as the resulting research data set was completely anonymous. The Geneva University Hospital Medical Ethical board approved this study and consent process: Authorization # 07-261R (NAC 07-102R).

### Study Design

**Figure 1:**
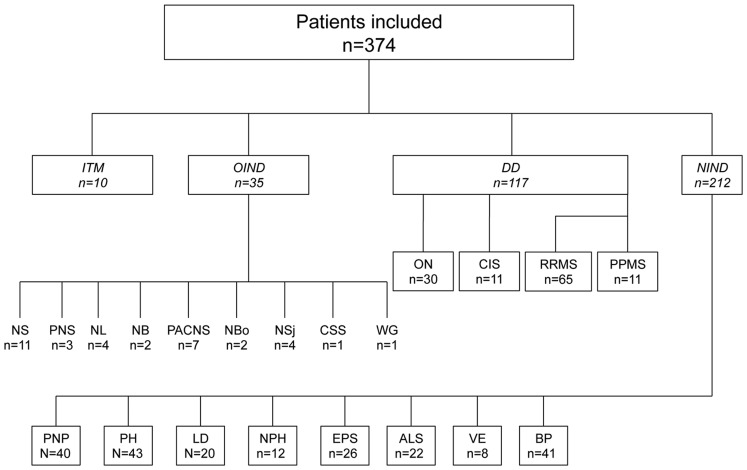
Description of the patient subgroups. ITM, idiopathic transverse myelitis; OIND, other inflammatory neurological diseases; NS, neurosarcoidosis; PNS, paraneoplastic syndrome; NL, neurolupus; NB, neuro-Behcet’s disease; PACNS, primary angiitis of the central nervous system; NBo, neuroborreliosis; NSj, neuro-Sjögren’s syndrome; CSS, Churg-Strauss syndrome; WG, Wegener granulomatosis; DD, demyelinating diseases; ON, optic neuritis; CIS, clinically isolated syndrome; RRMS, relapsing-remitting multiple sclerosis; PPMS, primary progressive multiple sclerosis; NIND, non-inflammatory neurological diseases; PNP, peripheral polyneuropathy; PH, primary headaches; LD, lumbar discopathy; NPH, normal pressure hydrocephalus; EPS, extrapyramidal syndromes; ALS, amyotrophic lateral sclerosis; VE, vascular encephalopathy; BP, Bell’s palsy.

This study included CSF samples from 374 patients representing 22 definite diagnoses and classified into four major diagnostic groups, which are summarised in Fig. 1. All 374 CSF specimens were obtained in the Division of Neurology at the Geneva University Hospital (Switzerland). We obtained 117 CSF samples from clinically ascertained patients with demyelinating diseases of the CNS: relapsing-remitting MS (RRMS) (n = 65), primary-progressive MS (PPMS) (n = 11), optic neuritis (ON) (n = 30), and clinically isolated syndrome (CIS) (n = 11). All CSF samples from MS and CIS patients were obtained during a relapse episode (i.e. within 4 weeks of a relapse, with the exception of PPMS). No patients with MS had received disease-modifying treatments up to the time of sample collection. All CIS patients were confirmed to have MS over time according to 2005 McDonald criteria. [14] Clinical and radiological characteristics of demyelinating disease (DD) patients are summarised in Tables 1 and 2. There was a 10-day maximal delay between MRI and spinal tap. Our study also included CSF samples from patients with idiopathic transverse myelitis (ITM, n = 10), as defined elsewhere, [15] and 35 patients with the following miscellaneous inflammatory neurological disorders (OIND) affecting the CNS: neurosarcoidosis (NS, n = 11), paraneoplastic syndrome (PNS, n = 3), neurolupus (NL, n = 4), neuro-Behcet’s disease (NB, n = 2), primary angiitis of the CNS (PACNS; n = 7), neuroborreliosis (NBo, n = 2), neuro-Sjögren’s syndrome (NSj, n = 4), Churg-Strauss syndrome with CNS involvement (CSS, n = 1), and Wegener’s granulomatosis with CNS involvement (WG, n = 1). We also obtained control CSF from 212 patients with the following non-inflammatory neurological diseases (NIND): polyneuropathy (PNP; n = 40), primary headaches (PH; n = 43), lumbar discopathy (LD; n = 20), normal pressure hydrocephalus (NPH; n = 12), extrapyramidal syndromes (EPD; n = 26), amyotrophic lateral sclerosis (ALS; n = 22), vascular encephalopathy (VE; n = 8), and Bell’s palsy (BP, n = 41).

**Table 1:** Clinical and biological characteristics of DD patients (n = 117).

	ON (n = 30)	CIS (n = 11)	RRMS (n = 65)	PPMS (n = 11)
Age (years), mean (± SD)	33.1 (±11.6)	37.4 (±14.2)	34.7 (±10.3)	52.7 (±12.6)
Female, n (%)	6 (20)	8 (73)	40 (62)	6 (55)
Disease duration (months), mean (± SD)	2.9 (±7.6)	2.2 (±2.2)	29.8 (±49.3)	122.3 (±150.8)
EDSS, mean (± SD)	N/A	1.8 (±0.8)	2.7 (±1.3)	3.4 (±1.5)
OB+(%)	13 (43)	9 (82)	59 (91)	10 (91)
CSF WBC>5 (%)	12 (40%)	5 (45%)	38 (60%)	2 (20%)

DD, demyelinating diseases; ON, optic neuritis; CIS, clinically isolated syndrome; RRMS, relapsing remitting multiple sclerosis; PPMS, primary progressive multiple sclerosis; SD, standard deviation; EDSS, expanded disability status scale; OB, oligoclonal bands; CSF, cerebrospinal fluid; WBC, white blood cells.

**Table 2:** Radiological characteristics of DD group subjects (n = 117).

	ON (n = 30)	CIS (n = 11)	RRMS (n = 65)	PPMS (n = 11)
**Brain MRI**	n = 18/30	n = 8/11	n = 55/65	n = 11/11
T1-Gd+ lesions, n (%)	5 (31)	0	19 (34)	0
Only T2 lesions, n (%)	6 (32)	4 (50)	36 (66)	11 (100)
No lesions, n (%)	7 (37)	4 (50)	0	0
**Spinal cord MRI**	n = 7/30	n = 7/11	n = 42/65	n = 11/11
T1-Gd+ lesions, n (%)	0	1 (14)	20 (48)	0
Only T2 lesions, n (%)	3 (43)	3 (43)	11 (26)	9 (82)
No lesions, n (%)	4 (57)	3 (43)	11 (26)	2 (18)

MRI, Magnetic Resonance Imaging; Gd, Gadolinium.

### Procedures

We used the revised 2005 McDonald Criteria [14] to diagnose MS. Three neurologists (AW, VK, and PHL) assigned final diagnoses to patients by reviewing all case histories and laboratory data, without knowledge of the serological results. We collected demographic and clinical information, including race, sex, age at onset, neurological symptoms and signs, history of autoimmunity, recording laboratory, and imaging data. CSF white blood cell (WBC) count and the determination of CSF-specific oligoclonal bands (OB) were retrieved from our CSF biobank database. OB testing was performed by electrophoresis and/or isoelectric focusing (IEF). CSF samples were obtained at the time of patients’ admission for routine diagnostic purposes. For MS patients, all lumbar punctures were made within 1 month of a relapse. CSF samples were stored at −80°C within 2 h of sampling. All CSF samples were tested in duplicate by an observer blinded to all clinical information. IL-6 measurement was performed with a quantitative and commercially available enzyme-linked immunosorbent assay (ELISA) according to the manufacturer’s instructions (R&D, # DY206). Results were expressed as pg/ml.

### Statistical Analysis

Owing to the asymmetric data distribution of CSF IL-6 levels, we applied non-parametric tests for statistical analysis. We used the Kruskal−Wallis test for the distribution analysis, and the Mann−Whitney U-test for the head-to-head comparisons. A p-value <0.05 was considered statistically significant. We used receiver operating characteristic (ROC) curve analysis to determine the power (sensitivity and specificity) of CSF IL-6 indices as a discriminatory diagnostic marker for MS. The area under the curve (AUC) was also evaluated. The Division of Clinical Epidemiology at the Geneva University Hospital (DC) performed all statistical analyses using SPSS (v. 18.0) statistical software.

## Results

**Figure 2:**
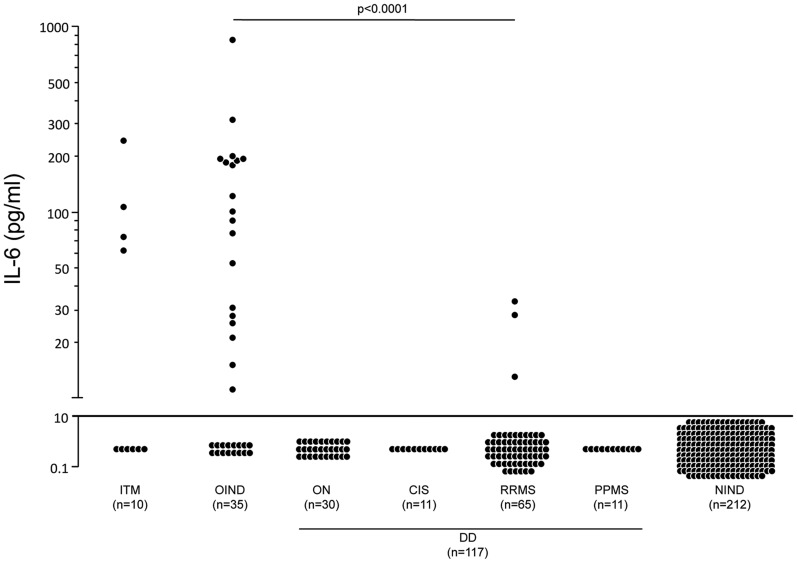
Distribution of CSF IL-6 levels in patient subgroups. The CSF IL-6 levels of all individual patients were measured using quantitative ELISA. Data are expressed as pg/ml and represented on a logarithmic scale. Only significant p-values are shown in the graph (p<0.001; Kruskal–Wallis test). The horizontal line shows the estimated optimal cut-off values for CSF IL-6 levels (10 pg/ml). ITM, idiopathic transverse myelitis; OIND, other inflammatory neurological diseases; ON, optic neuritis; CIS, clinically isolated syndrome; RRMS, relapsing-remitting multiple sclerosis; PPMS, primary progressive multiple sclerosis; NIND, non-inflammatory neurological diseases; DD, demyelinating diseases.

The levels of CSF IL-6 in different disease subgroups are shown in Fig. 2. We used ROC curves to determine the CSF IL-6 threshold value that would provide the best sensitivity and specificity as a discriminative criterion for clinical MS diagnosis. The cut-off value for CSF IL-6 was set at 10 pg/ml, yielding a 96% sensitivity to exclude MS at this value.

Given this cut-off value, CSF was IL-6−positive in 40% of patients with ITM (4/10; median: 4 pg/ml; interquartile range (IQR) 70) and 51% of patients with OIND (19/35; median: 15 pg/ml; IQR 111). By contrast, CSF was IL-6−positive in only 3.9% of MS patients (3/76; positive patients were all RRMS patients; median: 0 pg/ml; IQR 0.4), and in no patients with other DD, such as ON (median: 0 pg/ml; IQR 0.7) and CIS (median: 0 pg/ml; IQR 0.4). IL-6−positive CSF was not observed in patients with NIND (median: 0 pg/ml; IQR 0.2).

Head-to-head comparisons among the four different groups (ITM, OIND, DD, NIND; Fig. 2) indicated that results were statistically different between MS patients and patients with ITM (p<0.0001) or OIND (p<0.0001), but failed to reach significance between MS patients and patients with ON (p = 0.79) or NIND (p = 0.11). In particular, no CSF IL-6 levels greater than 7.6 pg/ml were observed in patients with NIND. In comparison, IL-6 reached levels as high as 250 pg/ml or 900 pg/ml in patients with ITM or OIND, respectively. We observed no IL-6 values greater than 33 pg/ml in patients with DD. We observed no differences among ON (n = 11), CIS (n = 11), RRMS (n = 65), and PPMS (n = 11) groups under scrutiny for CSF IL-6 index (p = 0.327). With the exception of three IL-6−positive samples, all in the RRMS group, we observed no IL-6 positivity in the CSF of DD patients (Fig. 2). Among the diseases of the OIND group, neuroborreliosis, Wegener disease, neurosarcoidosis and neurolupus have yielded the highest mean IL-6 levels. No statistically significant difference was found between the different subgroups (p = 0.66).

The clinical, biological, and radiological characteristics of DD patients are summarised in Tables 1 and 2. Notably, gadolinium-positive brain and spinal cord lesions, a classical hallmark of acute CNS inflammation in MS, were observed in 34% and 42% of RRMS patients, respectively. In addition, increased CSF WBC (>5 mm^3^) were observed in 60% of RRMS patients. Therefore, neither radiological nor biological patterns of inflammation observed in RRMS patients correlated with the observation of absent or low-level CSF IL-6.

It is noteworthy that the levels of IL-6 in the three IL-6−positive MS samples were found to be low (13 pg/ml, 28 pg/ml and 33 pg/ml, respectively) compared with ITM and OIND IL-6−positive samples. Two of the three IL-6−positive RRMS samples were associated with an elevated WBC CSF count above the average for the group (mean: 8 WBC). Two of them were also OB-positive. The brain MRI available for these three IL-6−positive MS patients revealed no T1 gadolinium-positive lesions; this was also the case regarding the single available spinal cord MRI. This finding indicates that the elevated CSF IL-6 levels observed in the three MS patients were not correlated with biological or radiological signs of CNS/CSF inflammation.

The proportion of positive CSF OB in RRMS and PPMS patients was 91%, which is compatible with observations of other MS cohorts. [16] Noteworthy, no OB were found in the samples of the OND group. In the OIND group, 18,7% of the samples were OB-positive.

We then compared the usefulness of OB versus IL-6 to discriminate MS patients. We observed that the absence of CSF IL-6 (96% sensitivity) was similar to the presence of OB (91% sensitivity) to confirm MS diagnosis. In addition, ROC curve analysis demonstrated that CSF IL-6 levels were more closely linked to MS than WBC counts, when the MS and OND groups were compared (data not shown). Altogether, these results strongly suggest that the measurement of IL-6 CSF levels as a quantitative assay could be useful to support a discriminatory clinical diagnosis of MS. It is however important to underline that OB testing mainly helps distinguishing between MS and healthy subjects or non-inflammatory neurological diseases, whereas IL-6 helps in our view in distinguishing MS and OIND.

## Discussion

The use of biomarkers is increasingly proposed as a method to refine the diagnosis and guide the treatment of numerous diseases. To date, no biomarker has had sufficient full-scale testing to qualify as a useful addition to standard diagnostic and prognostic factors, or to guide the prescription of specific treatments for MS. A recent study has shown promising results with the demonstration of the potassium channel KIR4.1 as a target of the autoantibody response in MS. [17] CSF OB detection remains currently the preferred biological test performed by physicians that investigate MS patients, although it is no longer required by the 2010 revised MS criteria. [13] When applying these revised criteria, it remains imperative that alternative diagnoses be considered and excluded. Thus, the identification of clinical and paraclinical red flags is of particular importance when diagnosing MS. Studies have investigated the possible usefulness of IL-6 as a biomarker of CNS diseases, especially in the context of demyelinating diseases; however, the small size of the studied samples and the inclusion of restricted neurological conditions limit its usefulness in clinical practice. We addressed this question in the present study by taking advantage of a large-scale data set that encompasses a variety of neurological conditions.

ROC curves, which represent the probability that a test will yield false-positive or false-negative results for all possible cut-off values, were drawn to determine the optimal cut-off value of CSF IL-6 to discriminate MS from OIND, a group of CNS inflammatory diseases considered in the classical differential diagnosis of MS. The results presented in this study indicate that elevated IL-6 levels (>10 pg/ml) are observed almost exclusively in CSF samples from patients with OIND and ITM, which likely reflects the inflammatory characteristics of the diseases represented in these groups. Thus, the measurement of CSF IL-6 with a cut-off of 10 pg/ml yielded a sensitivity of 96% to differentiate patients with MS from those with OIND of the CNS. Notably, these results have direct clinical implications, because ITM and OIND are typical diagnoses that may mimic MS and could therefore be mistaken for MS according to clinical presentation or MRI-restricted assessment. In contrast, CSF samples from patients with ON, CIS, MS, and NIND were negative for IL-6 reactivity, with the exception of three RRMS CSF samples that exhibited low IL-6 levels. Regarding the clinical and radiological characteristics of these patients, no difference with the other samples of the group was found. A satisfactory explanation for this low CSF IL-6 positivity is thus not available. In addition, separated analysis in patients with distinct DD revealed comparable results between the subgroups.

No single CSF test has proven fully reliable for distinguishing MS from OIND. Only CSF parameters such as CSF OB and IgG index are currently used to assist with MS diagnosis, although CSF analysis is no longer required for a diagnosis of MS. [13] In a hypothetical MS diagnosis, elevated CSF IL-6 values (i.e. >10 pg/ml) would indicate the need to investigate other possible diagnoses, especially in the spectrum represented here by the OIND group. Alternatively, CSF IL-6 measurement might be particularly relevant in cases of suspected clinical MS that are negative for CSF OB. In this regard, when MS is suspected in a patient with negative OB, the demonstration of IL-6 reactivity would strongly suggest the presence of another CNS inflammatory disease. Thus, the addition of a CSF IL-6 measurement combined with the detection of OB would permit a rapid discriminatory diagnosis in the context of MS.

IL-6 is a pleiotropic cytokine that is present during inflammation. [18] Elevated IL-6 levels are observed in almost every infectious and/or inflammatory condition. IL-6 is detectable in the CSF during viral, bacterial, and fungal infections of the CNS in humans. [19] Elevated IL-6 levels are also detectable in various diseases in which autoimmunity may play a role, such as rheumatoid arthritis [20] and lupus erythematosus. [21] In agreement with the results of our study, and despite the fact that MS is considered an autoimmune disease, little if any IL-6 has been detected in the CSF of MS patients. [19], [22], [23] To date, immunological markers of inflammation demonstrate little or no correlation with MS clinical disease progression, and there are few data on sufficiently long follow-up periods. [24] These results do not exclude the possibility that IL-6 is involved in MS pathogenesis. Indeed, IL-6 is produced locally in lesions and may not reach the CSF. The detection of IL-6 immunoreactivity in microglial cells and in reactive astrocytes in active MS plaques supports this hypothesis. [25] Surprisingly, IL-6 levels were elevated in the serum of MS patients, and were greater than levels in the CSF. [26] Nevertheless, our data suggest that IL-6 may not be primarily produced locally in the CSF of MS patients, or at least not released in the CSF compartment, in contrast to ITM or OIND.

In conclusion, we propose the measurement of CSF IL-6 as a relevant screen to discriminate MS patients from patients with OIND that may classically mimic MS. Although this test lacks specificity for any particular disease, the high IL-6 in the CSF renders the likelihood of MS less probable. In addition, this test could be easily performed on a routine basis in any center that is equipped to test CSF samples. Thus, we believe that this test should be considered as an added value to the CSF OB determination to help confirm or exclude MS.

## References

[1] Keegan BM, Noseworthy JH (2002) Multiple sclerosis. Annu Rev Med 53: 285–302. 11818475 10.1146/annurev.med.53.082901.103909

[2] Compston A, Coles A (2002) Multiple sclerosis. Lancet 359: 1221–1231. 11955556 10.1016/S0140-6736(02)08220-X

[3] Bielekova B, Martin R (2004) Development of biomarkers in multiple sclerosis. Brain 127: 1463–1478. 15180926 10.1093/brain/awh176

[4] Yuan X, Desiderio DM (2005) Proteomics analysis of human cerebrospinal fluid. J Chromatogr B Analyt Technol Biomed Life Sci 815: 179–189. 10.1016/j.jchromb.2004.06.044 15652808

[5] Romeo MJ, Espina V, Lowenthal M, Espina BH, Petricoin EF 3rd, et al (2005) CSF proteome: a protein repository for potential biomarker identification. Expert Rev Proteomics 2: 57–70. 15966853 10.1586/14789450.2.1.57

[6] Blennow K, Hampel H (2003) CSF markers for incipient Alzheimer’s disease. Lancet Neurol 2: 605–613. 14505582 10.1016/s1474-4422(03)00530-1

[7] Giovannoni G (2006) Multiple sclerosis cerebrospinal fluid biomarkers. Dis Markers 22: 187–196. 17124340 10.1155/2006/509476 PMC3851677

[8] Gadient RA, Otten UH (1997) Interleukin-6 (IL-6)–a molecule with both beneficial and destructive potentials. Prog Neurobiol 52: 379–390. 9304698 10.1016/s0301-0082(97)00021-x

[9] Pilcher H (2005) Protein culprit found for transverse myelitis? Lancet Neurol 4: 702. 10.1016/s1474-4422(05)70214-3 16265794

[10] Kaplin AI, Deshpande DM, Scott E, Krishnan C, Carmen JS, et al (2005) IL-6 induces regionally selective spinal cord injury in patients with the neuroinflammatory disorder transverse myelitis. J Clin Invest 115: 2731–2741. 16184194 10.1172/JCI25141 PMC1224298

[11] Ishizu T, Minohara M, Ichiyama T, Kira R, Tanaka M, et al (2006) CSF cytokine and chemokine profiles in acute disseminated encephalomyelitis. J Neuroimmunol 175: 52–58. 16697050 10.1016/j.jneuroim.2006.03.020

[12] Uzawa A, Mori M, Ito M, Uchida T, Hayakawa S, et al (2009) Markedly increased CSF interleukin-6 levels in neuromyelitis optica, but not in multiple sclerosis. J Neurol 256: 2082–2084. 19655191 10.1007/s00415-009-5274-4

[13] Polman CH, Reingold SC, Banwell B, Clanet M, Cohen JA, et al (2011) Diagnostic criteria for multiple sclerosis: 2010 revisions to the McDonald criteria. Ann Neurol 69: 292–302. 21387374 10.1002/ana.22366 PMC3084507

[14] Polman CH, Reingold SC, Edan G, Filippi M, Hartung HP, et al (2005) Diagnostic criteria for multiple sclerosis: 2005 revisions to the “McDonald Criteria”. Ann Neurol 58: 840–846. 16283615 10.1002/ana.20703

[15] Krishnan C, Kaplin AI, Deshpande DM, Pardo CA, Kerr DA (2004) Transverse Myelitis: pathogenesis, diagnosis and treatment. Front Biosci 9: 1483–1499. 14977560 10.2741/1351

[16] Freedman MS, Thompson EJ, Deisenhammer F, Giovannoni G, Grimsley G, et al (2005) Recommended standard of cerebrospinal fluid analysis in the diagnosis of multiple sclerosis: a consensus statement. Arch Neurol 62: 865–870. 15956157 10.1001/archneur.62.6.865

[17] Srivastava R, Aslam M, Kalluri SR, Schirmer L, Buck D, et al (2012) Potassium channel KIR4.1 as an immune target in multiple sclerosis. N Engl J Med 367: 115–123. 22784115 10.1056/NEJMoa1110740 PMC5131800

[18] Akira S, Taga T, Kishimoto T (1993) Interleukin-6 in biology and medicine. Adv Immunol 54: 1–78. 8379461 10.1016/s0065-2776(08)60532-5

[19] Houssiau FA, Bukasa K, Sindic CJ, Van Damme J, Van Snick J (1988) Elevated levels of the 26K human hybridoma growth factor (interleukin 6) in cerebrospinal fluid of patients with acute infection of the central nervous system. Clin Exp Immunol 71: 320–323. 3349651 PMC1541434

[20] Houssiau FA, Devogelaer JP, Van Damme J, de Deuxchaisnes CN, Van Snick J (1988) Interleukin-6 in synovial fluid and serum of patients with rheumatoid arthritis and other inflammatory arthritides. Arthritis Rheum 31: 784–788. 3260102 10.1002/art.1780310614

[21] Swaak AJ, van Rooyen A, Aarden LA (1989) Interleukin-6 (IL-6) and acute phase proteins in the disease course of patients with systemic lupus erythematosus. Rheumatol Int 8: 263–268. 2471249 10.1007/BF00270982

[22] Hauser SL, Doolittle TH, Lincoln R, Brown RH, Dinarello CA (1990) Cytokine accumulations in CSF of multiple sclerosis patients: frequent detection of interleukin-1 and tumor necrosis factor but not interleukin-6. Neurology 40: 1735–1739. 2234430 10.1212/wnl.40.11.1735

[23] Maimone D, Gregory S, Arnason BG, Reder AT (1991) Cytokine levels in the cerebrospinal fluid and serum of patients with multiple sclerosis. J Neuroimmunol 32: 67–74. 2002092 10.1016/0165-5728(91)90073-g

[24] Harris VK, Sadiq SA (2009) Disease biomarkers in multiple sclerosis: potential for use in therapeutic decision making. Mol Diagn Ther 13: 225–244. 19712003 10.1007/BF03256329

[25] Maimone D, Guazzi GC, Annunziata P (1997) IL-6 detection in multiple sclerosis brain. J Neurol Sci 146: 59–65. 9077497 10.1016/s0022-510x(96)00283-3

[26] Frei K, Fredrikson S, Fontana A, Link H (1991) Interleukin-6 is elevated in plasma in multiple sclerosis. J Neuroimmunol 31: 147–153. 1991821 10.1016/0165-5728(91)90020-8

